# The role of the Chief Health Strategist in community health improvement: a MAPP 2.0 counterproposal

**DOI:** 10.3389/fpubh.2025.1601406

**Published:** 2025-07-09

**Authors:** Angela L. Carman, Mary Elizabeth Pendergrass

**Affiliations:** ^1^Health Management and Policy, University of Kentucky College of Public Health, Lexington, KY, United States; ^2^Public Health Policy and Practice Apprentice, University of Kentucky College of Public Health, Lexington, KY, United States

**Keywords:** Chief Health Strategist, community health improvement, trust, decision making, community partners

## Abstract

In the past 10 years, several significant national initiatives have released updated strategies and guidelines to improve public health practice specifically as it relates to community health improvement. These initiatives include the 2016 release of Public Health 3.0 by the U.S. Department of Health and Human Services (HHS) Office of the Assistant Secretary for Health (OASH), which defines the concept of the Chief Health Strategist as a leader in the community’s health improvement efforts. In addition, in 2022, the Public Health Accreditation Board (PHAB) released guidance for community health improvement through a revised set of accreditation standards and measures, which included a list of suggested models such as Mobilizing for Action through Planning and Partnerships (MAPP) to guide the process. Finally, a revised model, MAPP 2.0, was released in 2023 to provide updates to the National Association of County and City Health Officials’ (NACCHO’s) original framework for community health improvement. Despite the valuable information for a collaborative approach to community health improvement found in the 2022 PHAB accreditation standards and measures and in MAPP 2.0, the role of the Chief Health Strategist from Public Health 3.0 is missing. This article describes the importance of the role of the Chief Health Strategist in community health improvement, emphasizing community trust-building, the ability to galvanize community group participation, and the use of systems thinking and decision-making to create a counterproposal to the guidelines presented in MAPP 2.0.

## 1 Community health improvement

### 1.1 Introduction

The community health improvement planning process involves collaboration between governmental public health organizations and organizations that directly (e.g., hospitals and clinics) or indirectly (e.g., non-profits, employers, housing providers, and emergency services) affect health in a specific community (1). In 2011, the Public Health Accreditation Board (PHAB) launched its first set of accreditation standards, including a process built around continuous quality improvement and the 10 Essential Public Health Services (2–4) that required state, local, tribal, and territorial health departments pursuing accreditation to complete a Community Health Assessment (CHA) and Community Health Improvement Plan (CHIP) (1, 5). The completed CHA/CHIP document, developed following PHAB guidelines, includes a list of community partners included in the process, data from the Community Health Assessment, and selected community focus areas with measurable goals and objectives (1, 5–8).

Many other national organizations have released guidelines and competencies to support the completion of high-quality, comprehensive Community Health Assessments and Improvement Plans. One such organization, the National Association of City and County Health Officials (NACCHO), works closely with Local Health Departments (LHDs) to develop practice tools and guidelines. NACCHO’s 2022 Profile of Local Health Departments indicates that, in the past 5 years, 65% of local health departments (LHD) had completed a CHIP (9, 10). Studies conducted on the CHIPs produced by LHDs have often shown that medium and large LHDs are most likely to have participated in a recent (last 5 years) CHA/CHIP (9–11) and to have used the Mobilizing Action through Planning and Partnerships (MAPP) model as a guide (9, 10, 12).

Public Health 3.0 is another initiative from a listening tour of public health leaders, released by the U.S. Department of Health and Human Services (HHS) Office of the Assistant Secretary for Health (OASH). It outlines the attributes and responsibilities of the Chief Health Strategist, many of which coincide with recommendations from PHAB and NACCHO that partnerships and collaboration are essential aspects of effective community health planning (8, 13, 14).

Previous studies also support that the most frequently identified CHIP focus areas or strategies dealt with service provision and education on specific health topics such as chronic disease, nutrition, and physical activity (6, 10). In addition, some CHIPs identified areas for potential growth in the community in partnership development and advocacy (10). These findings, alongside the frequent use of educational strategies, align with Frieden’s Health Impact Pyramid, which suggests that the health improvement strategies easiest to implement (e.g., addressing education and training) tend to have the least impact on public health (15). Conversely, strategies addressing the Social Determinants of Health (SDOH) are the hardest to implement but can have the greatest impact at the population level (6, 10, 15, 16).

However, the literature supports addressing issues defined by the CDC (17), such as the Social Determinants of Health (SDOH), to more comprehensively and sustainably improve health by addressing the root cause (6). These elements—Education Access and Quality, Healthcare and Quality, Neighborhood and Built Environment, Social and Community Context, and Economic Stability—direct community health improvement planning toward the “upstream” factors that contribute to health outcomes, rather than focusing solely on resulting conditions or diseases (17). Multiple studies confirm that while LHD CHIPs may acknowledge SDOH, few include well-constructed strategies for improvement (6, 12, 18, 19). This lack of strategies to address SDOH in LHD CHIPs may stem from limited resources and/or insufficient data on these complex issues (7, 11). In addition, a lack of focus on SDOH may result from issues within the collaborative process of bringing partners together. Without the right partners at the table to champion specific SDOH, the discussion may lag and remaining partners often determine that these issues are outside of their sphere of influence (11, 20, 21). This study includes a review of the role of the Chief Health Strategist as introduced in Public Health 3.0 in community health improvement, the standards and practice of community health improvement as specified by the Public Health Accreditation Standards, and the MAPP 2.0 model for participatory community health improvement planning and a counterproposal to the MAPP 2.0 model which substantiates, through published literature, the impact the Chief Health Strategist could have on the process.

### 1.2 Public health 3.0 and the role of the Chief Health Strategist in community health improvement

In 2016, the U.S. Department of Health and Human Services (HHS) Office of the Assistant Secretary for Health (OASH) initiated a listening tour with diverse local leaders in public health and other related sectors to discuss health promotion efforts and share strategies for moving public health forward (22, 23). The resulting report, Public Health 3.0, builds on past accomplishments of public health and adds five recommendations:

1. “Strong leadership and workforce
2. Strategic partnerships
3. Flexible and sustainable funding
4. Timely and locally relevant data, metrics, and analytics
5. Foundational infrastructure” (23).

In recommendation #1, Strong leadership and workforce, the concept of the Chief Health Strategist is introduced. Public Health 3.0 defines the Chief Health Strategist as a leader in community health promotion efforts in collaboration with partners from healthcare and various other diverse sectors to address issues of prevention and wellness (23, 24). In addition, a critical role of the Chief Health Strategist is to convene and encourage participation from other community leaders in the task of health improvement and implementation of community health improvement plans, specifically as they relate to the “upstream” SDOH (23, 24). The Community Chief Health Strategist Competencies are detailed by NACCHO (See Table 1).

**Table 1:** NAACHO Chief Health Strategist competencies (25).

Community health strategist competency	Description
Practice #1	Adopt and adapt strategies to combat the evolving leading causes of illness, injury, and premature death.
Practice #2	Develop strategies for promoting health and wellbeing that work most effectively for communities of today and tomorrow.
Practice #3	Identify, analyze, and distribute information from new, big, and real-time data sources.
Practice #4	Build a more integrated, effective health system through collaboration between clinical care and public health.
Practice #5	Collaborate with a broad array of allies, including those at the neighborhood-level and the non-health sectors, to build healthier and more vital communities.
Practice #6	Replace outdated organizational practices with state-of-the-art business, accountability, and financing systems.
Practice #7	Practice #7—Work with corresponding federal partners—ideally, a federal Community Chief Health Strategist—to effectively meet the needs of their communities.

Public Health 3.0 describes the Chief Health Strategist in many communities as the local health officer while acknowledging that other sectors within the community may also host leadership roles (23). However, the document from NACCHO, which describes the Chief Health Strategist competencies, identifies the local public health *department* as the Chief Health Strategist (25). This small but important detail identifies the Chief Health Strategist as a leader in community health improvement and emphasizes the local health department as a critical organization, comprising multiple functions, whose focus on prevention and wellness is a vital component of community health improvement efforts.

After the release of Public Health 3.0, feedback from public health practitioners was sought, which resulted in the following “call to action” for public health organizations:

1. “Adopt the Chief Health Strategist model
2. Establish structured, cross-sector partnerships
3. *Seek accreditation*
4. Acquire actionable data and establish clear metrics
5. Enhance and de-silo funding for public health” (26).

### 1.3 National voluntary public health accreditation—setting standards for community health improvement

Following the identification of the Chief Health Strategist in Public Health 3.0 and the “call to action” by public health practitioners to embrace this role was also a call to seek public health accreditation. “The Future of Public Health in the 21st Century,” released in 2002 by the Institute of Medicine, recommended that public health accreditation be explored as a means to improve the performance of public health departments (27). The Public Health Accreditation Board (PHAB) launched the first voluntary accreditation system in 2011, establishing consensus standards and measures for state, local, tribal, and territorial public health agencies in the United States (2). These initial accreditation standards placed significant emphasis on the completion of the Community Health Assessment and the Community Health Improvement Plan by any local health department seeking accreditation (2). As of March 2025, the public health accreditation board lists 41 state, 400 local, and 6 tribal health departments as accredited (28).

The 2022 release of the Public Health Accreditation Board’s (PHAB) revised standards and measures for public health accreditation (13) provided updated guidelines for community health assessment (CHA) and the development of a community health improvement plan (CHIP) for public health organizations responding to the “call to action” to seek accreditation (13, 26). Specifically, in Domain 7 of the PHAB standards and measures, Standard 7.1 directs health departments to “Engage with partners in the healthcare system to assess and improve health service availability” (13). This standard provides an example of the Chief Health Strategist’s role, that is, engagement with community partners as a critical responsibility (13).

PHAB’s 2022 Standard 1.1 directs health departments to “Participate in or lead a collaborative process resulting in a comprehensive community health assessment (CHA)” (13). In the guidance provided by PHAB for conducting the CHA, health departments are given examples of national models that provide processes for collaborating with community partners. Mobilizing Action through Planning and Partnerships (MAPP) is listed as one of the community-engaged models to guide the CHA/CHIP process (8, 13).

### 1.4 Mobilizing action through planning and partnerships 2.0—a guide for community health improvement practice

MAPP was developed by the National Association of County and City Health Officials (NACCHO) and the Public Health Practice Program Office of the Centers for Disease Control and Prevention (CDC) and released in 2002 (29). The MAPP development workgroup intended for this tool to be used by communities as a performance improvement initiative through which effective local leadership (30) could engage the community through all phases of the project, thereby building trust across the members of a community’s local public health system (18). This focus on the role of leadership in the MAPP process is ideally suited to Public Health 3.0’s definition of the Chief Health Strategist role (22, 23). Phases of the original MAPP model began with Organizing for Success/Partnership Development and Visioning, followed by four assessments—Community Themes and Strengths Assessment, Local Public Health System Assessment, Community Health Status Assessment, and the Forces of Change Assessment—and then proceeded to Identifying Strategic Issues, Formulating Goals and Strategies, and Implementation (31). The 2002 NACCHO National Profile for Local Health Departments states that approximately one in four LHDs who completed a CHIP in the last 5 years used MAPP (9).

In the study of MAPP demonstration sites by Pullen et al., the activity levels of organizational leadership during the MAPP process were identified (29). MAPP sites with low activity levels for organizational leadership activity were described as isolated within an area of the health department with community members and organizations not understanding how they could or should be involved (29). In contrast, high activity levels for organizational leadership demonstrated strong buy-in by the health department director and designated full-time staff for the MAPP process, both of which were deemed integral to the development of strong community partnerships (29).

Additional studies of the MAPP process have identified issues that could limit the effectiveness of the community health improvement process model. Limited knowledge or experience with public health of those guiding the process (26), the complexity and time required to execute MAPP tools (32), and poorly organized MAPP meetings leading to members having “meeting burnout” (33) could potentially reduce the desired collaborative efforts to improve community health.

Mobilizing Action through Planning and Partnerships 2.0 (MAPP 2.0) was released in 2023 to provide updates to the CHA/CHIP process (8). The initial phase of Organizing for Success/Partnership Development is relaunched in MAPP 2.0 as Phase 1: Building the Community Health Improvement (CHI) Foundation. This phase provides tools for assessing Stakeholders and Power Analysis, a Starting Point Assessment for resource inventory, and a vision for MAPP (8). This phase and the tools provided are intended to build relationships that lead to commitment and ownership by the community to the process (8).

A significant change in the MAPP 2.0 version is found in the steps that guide the assessment of health within a specific community (8). Prior to the MAPP 2.0 release, community health assessments included The Local Public Health System Assessment (LPHSA) (34), which guided participants in a discussion of the 10 Essential Public Health Services (4), including where each was active in the community. The MAPP 2.0 version changed the LPHSA to a Community Partner Assessment, which is a tool designed for community partners involved in MAPP to critically examine their individual systems, processes, and capacities and the collective capacity of the group of community partners (35). The training manual for MAPP 2.0 instructs, for the Community Partner Assessment only, that partners should be prepared to spend 14.5–17.5 h on this assessment and related training and discussions.

## 2 MAPP 2.0 counterproposal

### 2.1 Establish the importance of the Chief Health Strategist

The guidelines for MAPP 2.0 (8) and specifically the Community Partner Assessment (CPA) (35) provide valuable information for a collaborative approach to community health improvement. Local health departments that use these guidelines are not only completing a required step in the public health accreditation process (13) but are also potentially building a community health improvement plan that includes the critical voices of community members. However, despite the updates to the MAPP 2.0 process and the 2022 PHAB standards and measures, an explicit description of the Chief Health Strategist role from Public Health 3.0 is missing. Leadership roles for MAPP 2.0 Phase 1: Building the CHI Foundation, specifically for individual assessments within MAPP such as CPA are described as “CPA leads” or a “point person” (8). However, the incredibly important work of building relationships and trust, galvanizing community participation in health improvement, and guiding strategic thinking and decision-making throughout the CHI process is not specifically assigned. This article’s counterproposal to the MAPP 2.0 model for Community Health Assessment and Community Health Improvement Planning (CHA/CHIP) focuses on the assignment of the Chief Health Strategist as the leader of the CHA/CHIP process due to the ability of this leadership position, as substantiated in literature, to build trust, galvanize community member participation, and provide strategic thinking and decision-making.

#### 2.1.1 Building trust

Leaders who interact both inside and outside of their own organizations with a desire to listen and learn from others can initiate the trust-building process (36, 37). By understanding the general happenings of the community and the specific needs of partners, a leader, such as the Chief Health Strategist, can identify points of mutual needs or potential connections between community organizations (38). Although, the trust-building process may take time, particularly when distrust exists or past experiences of working together have been unsuccessful (38). However, numerous community projects detailed in the literature highlight how positive experiences working with a specific leader significantly contribute to positive outcomes (7, 11, 20, 32, 39–42). The impact of a Chief Health Strategist who is trusted by community members, specifically other leaders and decision-makers, is the beginning of a powerful community connection. These trusting connections can effectively engage other leaders and decision-makers in the community health improvement process (43).

#### 2.1.2 Galvanize community member participation

A quote from Nicola and Hatcher’s article “A Framework for Building Public Health Constituencies” makes a powerful statement in the context of partnerships for community health improvement: “Knowing the community and its constituents is more than an epidemiological assessment” (44). To truly know the community, and the potential partners within it, a Chief Health Strategist continuously networks with other leaders and residents in a community, asks questions, and listens and learns as discussed in the Building Trust section 2.2.1.

The Chief Health Strategist also leans into Competency #3, regarding uses of data, and #6, which discusses new ways of doing things, to understand which populations within the community are impacted by specific concerns and help to identify new ways of addressing those concerns. Adding these competencies to those that build on trusting relationships can bring people together to truly improve their community. Another quote from Nicola and Hatcher states “If public health leaders view networking as an ongoing and essential activity in the agency’s operations, constituency mobilization can be productive and require minimal effort” (44). Networking is so much more than just chatting with people: it involves understanding their perspectives, seeing where they live and work, and offering some of a leader’s time to deepen understanding (37).

#### 2.1.3 Strategic thinking and decision-making

The Chief Health Strategist, whose competencies focus heavily on collaboration and strategic thinking (25), is also ideally suited to maximize the elements of their leadership position to draw in community members to the community health improvement effort. Leadership and management literature details the components of leadership power (45). Two specific types of leadership/management power relevant to the Chief Health Strategist are legitimate power and expert power (45). Legitimate power focuses on the position held by a particular leader and includes the authority levels of that position (45, 46). For example, a local health department leader/director has the formal authority or legitimate power to commit the health department to participate in a specific community effort or to assign human resources to specific tasks and opportunities. In addition, the Chief Health Strategist, when fully meeting and maximizing the Chief Health Strategist competencies (25), has special knowledge in community health improvement and working through collaborations and thus can wield expert power (45). The same levels of legitimate and expert power exist with other leaders in the community such as school superintendents, city planners, healthcare leaders, and non-profit leaders. These are the community decision-makers with whom the Chief Health Strategist must network.

Considering the legitimate and expert power of a Chief Health Strategist together with human and conceptual management and leadership skills, skills of effective leaders have been studied as part of management literature for many years. Specifically, human skills are defined as “the executive’s ability to work effectively as a group member and to build cooperative effort within the team he leads—Primarily concerned with working with people” and are required at all levels of management (47, 48) but are especially critical for the Chief Health Strategist. Mastery of these human skills of management enables the Chief Health Strategist to achieve competencies #4, 5, and 7, which focus on galvanizing partnerships to work together (25).

Conceptual skills are problem-solving skills through which a leader understands relationships between parts of a system and the impact of change in any of those parts on the others (45). These conceptual skills are necessary to uncover and address multifaceted problems (33). considering the combination, in the Chief Health Strategist, of the collaborative human skills, conceptual problem-solving skills, legitimate power of their position, plus the expert power that comes with mastery of Chief Health Strategist competencies #1, 2, 3, and 6 (25, 45, 47), the result is an influential champion for community health improvement (49–51). This leader can bring people together, problem-solve, and, together with other community leaders, has the power to make decisions and implement them.

To delegate the important work of gathering community voices and leading a community health improvement project to someone other than the Chief Health Strategist is to fail to maximize what this leader is and what they bring to the effort. Accordingly, MAPP 2.0 must explicitly assign the Chief Health Strategist role as a defined part of the CHI process. Through this designation, health departments utilizing the MAPP 2.0 model will understand the importance of the Chief Health Strategist’s role and thus benefit from its inclusion in the process.

### 2.2 Reorganize specific MAPP 2.0 tools and assessments as Chief Health Strategist guidelines

This article’s counterproposal to specifically assign the Chief Health Strategist as the leader of the MAPP 2.0-guided CHA/CHIP process allows for the continued use of MAPP 2.0 tools and assessments but is reorganized as guidelines and suggestions for the Chief Health Strategist to use while working with the community. MAPP 2.0 includes several assessment tools to guide those involved in the process through the collection of important information (see Table 2). The Community Partner Assessment matches well with the previously discussed elements of building trust and galvanizing community member participation in the MAPP 2.0 process. This assessment involves several in-person partner meetings, as well as a 59-question survey. A sample of questions that MAPP 2.0 suggests for the survey includes the following:

- “Q6—What are your organization’s top three interests in joining a community health improvement partnership?
- Q18—Who are your priority populations?
- Q20—Does the leadership of your organization reflect the demographics of the community you serve?
- Q47—What data skills does your organization have? (35).”

**Table 2:** MAPP 2.0 three assessments (8).

Assessment	Data used	Purpose
Community status assessment	Quantitative data	Help community understand “upstream” factors, root causes, and SDOH.
Community context assessment	Qualitative data	Understand strengths, assets, and change elements at play in the community.
Community partner assessment	Quantitative and qualitative data	Allows community partners to look at their organizational capacity and to determine the collective capacity in the community.

In addition, the MAPP 2.0 training manual suggests that LHDs should request the following from partners in terms of time commitment for the Community Partner Assessment:

- First Orientation Meeting: 2–2.5 h
- Second Orientation Meeting–2-2.5 h
- Survey: 30–40 min
- Partner discussion: 10–12 h
- Total: 14.5–17.5 h (8).

This time commitment is for one assessment tool only. If trust and interest in the community health improvement planning process have not been appropriately developed, it is possible that community partners might find this excessive. If the Chief Health Strategist operates according to NAACHO’s competencies, much of the information gathered through the CPA could be adapted as a guide to support the Chief Health Strategist in developing and maintaining relationships. As part of their ongoing role in community networking, the Chief Health Strategist would naturally gather information about partners, potentially reducing the burden on those partners to invest significant time answering organizational questions.

### 2.3 Designate a MAPP 2.0 backbone support structure to support the Chief Health Strategist

**Figure 1:**
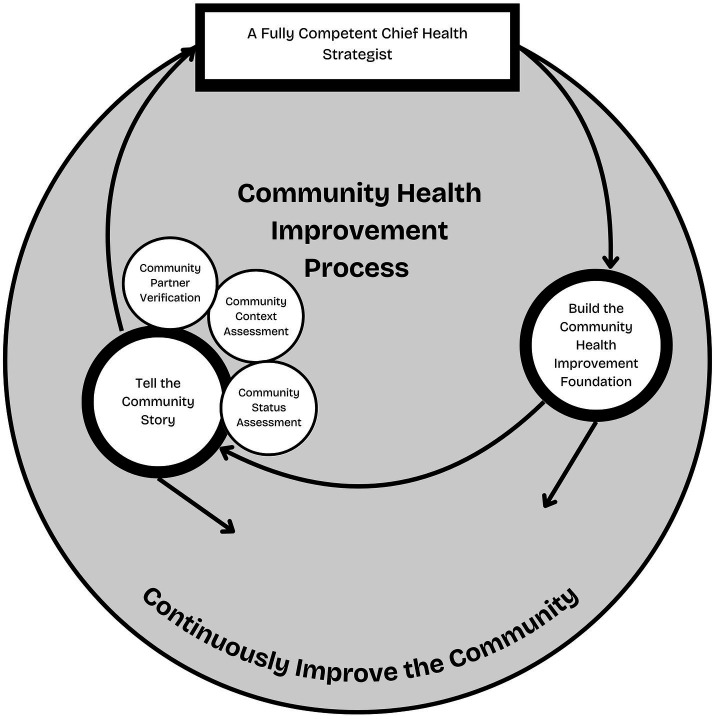
Incorporation of Chief Health Strategist community health improvement process.

In Section 2.2, a counterproposal is presented that shifts the role of community partners in completing assessments under MAPP 2.0, instead proposing that the assessments serve as guides for the Chief Health Strategist. This counterproposal is made with full awareness of the amount of work for which the Chief Health Strategist is responsible. It would be unrealistic to assume that the Chief Health Strategist would be able to complete all elements required of a community health improvement process; thus, the development of the MAPP 2.0 Backbone Support structure, within the Chief Health Strategist’s organization, is suggested to support the Chief Health Strategist’s role in the MAPP 2.0 Counterproposal. Figure 1 depicts a revised MAPP 2.0 process, including the Chief Health Strategist.

The Chief Health Strategist should design a structure within their organization to support their leadership of the critical process of assessing the health of their community and collaboratively designing a community health improvement plan with coalition members (44, 52). The work of the Chief Health Strategist to build trust and galvanize community member participation in community health improvement will be time-consuming and require assistance from inside the organization with administrative details of the work. Specifically, from the five Conditions of Collective Impact, the backbone support structure is recommended. A Backbone Support Structure dedicates human resources to coordinate elements of the CHI process and the communication required for partners to understand what is required (49). This recommendation is made for a Backbone Support Structure within the Chief Health Strategist’s organization. Staff members would require training in the Backbone Support concept from Collective Impact, participatory planning as outlined in the MAPP model (8), and community health improvement. They should also be trained to follow the Chief Health Strategist’s lead when interacting with partners (22, 53, 54). These dedicated human resources would allow the Chief Health Strategist to lead the important work on community health assessment and community health improvement planning.

## 3 Conclusion

As stated through recommendations from national organizations, such as PHAB and NACCHO, and initiatives such as Public Health 3.0 and MAPP, partnerships and collaboration are essential to the CHI process. Specifically, collaboration among a variety of community partners has the best chance to impact factors related to root causes and the Social Determinants of Health.

Although MAPP 2.0 recognizes the importance of community collaboration and devotes time through the Community Partner Assessment to this collaboration, the result is a snapshot of potential community partners and their capabilities. This snapshot information can be valuable, but an underlying assumption exists that the community partners, needed to address multi-faceted problems, agreed to come to the table and complete the assessment. Without the previously recommended trust built by a Chief Health Strategist engaged in the CHI process, the partners may not have identified this process as an appropriate use of their time. In addition, without the work of a Chief Health Strategist to galvanize community partnerships with other leaders in the community who also wield legitimate and expert power and have experience with strategic thinking and decision-making, the community representatives at the table for the MAPP process may not have the authority or power to commit their organizations to specific actions needed to address aspects of the community’s health.

To achieve effective community collaboration necessary for strategic community health improvement, the MAPP 2.0 process, due to its use by many LHDs and their communities, must involve an explicitly stated role for the Chief Health Strategist. Thus, through the impact of the Chief Health Strategist in building trust, galvanizing community participation, and promoting strategic thinking and decision-making, NACCHO’s MAPP 2.0 model can establish effective community collaboration and, thus, health improvement may be achieved.
